# CKS1B promotes cell proliferation and invasion by activating STAT3/PD‐L1 and phosphorylation of Akt signaling in papillary thyroid carcinoma

**DOI:** 10.1002/jcla.23565

**Published:** 2020-09-22

**Authors:** Hui Wang, Zhengdong Zhang, Zhe Yan, Shihong Ma

**Affiliations:** ^1^ Shanghai Xuhui Center Hospital Shanghai China

**Keywords:** Akt, CKS1B, papillary thyroid carcinoma, STAT3/PD‐L1

## Abstract

**Objective:**

To investigate role of GKS1B and its relationship between STAT3/PD‐L1 and p‐Akt in papillary thyroid carcinoma (PTC).

**Methods:**

Expression of GKS1B and PD‐L1 was determined in PTC cell lines. GKS1B was overexpressed or knocked down by transfection with overexpression plasmids or si‐CKS1B. STAT3 inhibitor WP1066 was used to suppress STAT3, and PD‐L1 inhibitor Pembrolizumab was used to block PD‐L1. Cell viability and invasion were evaluated by MTT and transwell assay, respectively. The expression of STAT3, p‐STAT3, Akt, and p‐Akt was measured using Western blotting.

**Results:**

Both protein levels and mRNA levels of CKS1B and PD‐L1 were remarkably up‐regulated in PTC cell lines. Knockdown of CKS1B significantly inhibited cell viability and invasion of PTC cells and suppressed STAT3/PD‐L1 signaling and Akt phosphorylation, while overexpression of CKS1B led to opposite results. Inhibition of STAT3 or PD‐L1 reversed the effects of overexpressed CKS1B on PTC cells.

**Conclusion:**

The overexpression of CSK1B could promote cell viability and invasion of PTC cells through activation of STAT3/PD‐L1 signaling and Akt phosphorylation.

## Graphical abstract

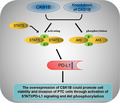

## 1 INTRODUCTION

Papillary thyroid carcinoma (PTC) is the most common type of thyroid carcinoma which accounts for approximate 85%~70% cases of thyroid carcinoma (PTC).^1^, ^2^ As a kind of differentiated thyroid carcinoma (DTC), PTC patients always have better prognosis with slighter malignance.^3^, ^4^ However, prognosis for patients with recurrence and metastasis is still poor, with a 5‐year survival rate only about 50%.^5^, ^6^ Despite numerous studies on PTC, the molecular mechanisms for PTC development are still unclear.

CDC28 protein kinase regulatory subunit 1B (CKS1B), a member of CKS family, can bind to the catalytic subunit of CDK to regulate cell cycle function.^7^ Recent researches found it is a cancer promoter which can facilitate cancer development or is associated with poor prognosis of cancer patients in oral squamous cell carcinoma,^8^ nasopharyngeal carcinoma,^7^ and myeloma.^9^ However, role of CKS1B in PTC is rarely reported.

Signal transducer and activator of transcription 3 (STAT3) is a widely known protein which plays important roles in many diseases, including cancer development.^10^, ^11^ Recent studies found CKS1B could regulate STAT3 and further influence cancer development of lung cancer and myeloma.^12^, ^13^ Besides, other studies showed STAT3 is an upstream protein of programmed death ligand 1 (PD‐L1), also known as CD274.^12^, ^14^ And in a recent research, Lubin et al demonstrated PD‐L1 was elevated in PTC and was related to patients’ prognosis.^15^ However, up to now, no study focused on the relationship between CSK1B and STAT3/PD‐L1 signaling in PTC.

In the present study, we demonstrated that the overexpression of CSK1B could promote cell viability and invasion of PTC cells through activation of STAT3/PD‐L1 signaling and Akt phosphorylation. This study might provide deeper understanding of CSK1B/STAT3/PD‐L1 axis in PTC development.

## 2 METHODS AND MATERIALS

### 2.1 Cell culture and transfection

PTC cell lines W3, TPC1, IHH‐4, BCPAP, and K1 cells, as well as normal thyroid cell line Nthy‐ori‐3 cells, were purchased from ATCC (Manassas, VA, USA). Cells were cultured in glucose‐deficient RPMI 1640 medium containing 10% Gibco^®^ fetal bovine serum (FBS) and 100 μg/mL penicillin‐streptomycin (Sigma‐Aldrich Co, USA) at 37°C and 5% CO_2_. After being cultured to 70 ~ 80% confluence, cells were transfected with si‐CKS1B or si‐NC (designed and synthesized by Shanghai GenePharma Co., Ltd), as well as overexpression plasmids for CKS1B (OE‐CKS1B, designed and synthesized by Shanghai GenePharma Co., Ltd) and the negative control (NC) using the Lipofectamine 3000 (Invitrogen) in serum‐free Opti‐MEM medium according to the manufacturer's instruction. Transfection efficacy was determined after 48 hours of surgery. For inhibiting STAT3, STAT3 inhibitor WP1066 (5 μmol/L, Sigma‐Aldrich) was used to treat the cells. For inhibition of PD‐L1, PD‐L1 inhibitor Pembrolizumab (5 μmol/L, Merck Sharp & Dohme Corp) was used to treat the cells.

### 2.2 MTT assay

MTT assay was used for evaluation of cell viability. Briefly, cells (3 × 10^4^) seeded in 96‐well plates were cultured for 48 hours under 37°C and 5% CO_2_. After addition of 10 μl MTT solution (5 mg/mL), cells were cultured for another 4 hours. Then, 150 μL DMSO was added after removing MTT, and the value of optical density (OD) was evaluated 490 nm.

### 2.3 Transwell assay

Transwell assay was used for determination of cell invasion. For invasion, 1 × 10^5^ cells were plated in the top chamber with Matrigel‐coated membrane (24‐well insert; pore size, 8 mm; BD Biosciences). Cells were then cultured for 24 hours in serum‐free media. Then, cells were stained with 0.1% crystal violet. The invaded or migrated cells were counted and photographed using light microscope (Zeiss, Germany). The invaded or migrated cell rates were calculated as the invaded or migrated cell number in the tested group/blank control group.

### 2.4 Reverse transcription‐quantitative polymerase chain reaction (RT‐qPCR)

The expression levels of CKS1B and PD‐L1 were evaluated using qRT‐PCR. Total RNA was extracted using the Trizol reagent (Tiangen Biotech, Beijing, China). After converting RNA to cDNA by a High‐Capacity cDNA Reverse Transcription kit (Applied Biosystems), the PCRs were then performed in Applied Biosystems 7500 Real‐Time PCR System (Applied Biosystems) using SYBR GREEN mastermix (Solarbio, Beijing, China) in an Exicycler™ 96 (Bioneer). Relative RNA levels were calculated by the 2^‐ΔΔCq^ method. GAPDH was used as an internal control.

### 2.5 Western blotting

The protein levels of CKS1B, STAT3, p‐STAT3, PD‐L1, Akt, and p‐Akt were determined using Western blotting. The extracted proteins were loaded on 10% SDS‐PAGE, transferred to PVDF membranes, and blocked using 5% non‐fat milk. Samples were then incubated with the following specific primary antibodies (Abcam) at 4°C overnight. After being incubated with corresponding secondary antibody at 37˚C for 45 min, protein bands were analyzed with the Pierce ECL Western Blotting Substrate (Pierce, Shanghai, China). GAPDH was served as an internal control.

### 2.6 Statistical analysis

The measurement data were expressed by mean ± SD. Comparisons were made using one‐way analysis of variance (ANOVA) followed by Tukey post hoc test. It was considered to be statistically significant when P‐value was less than 0.05. All calculations were made using SPSS 22.0.

## 3 RESULTS

### 3.1 CKS1B and PD‐L1 were highly expressed in PTC cell lines

**Figure 1:**
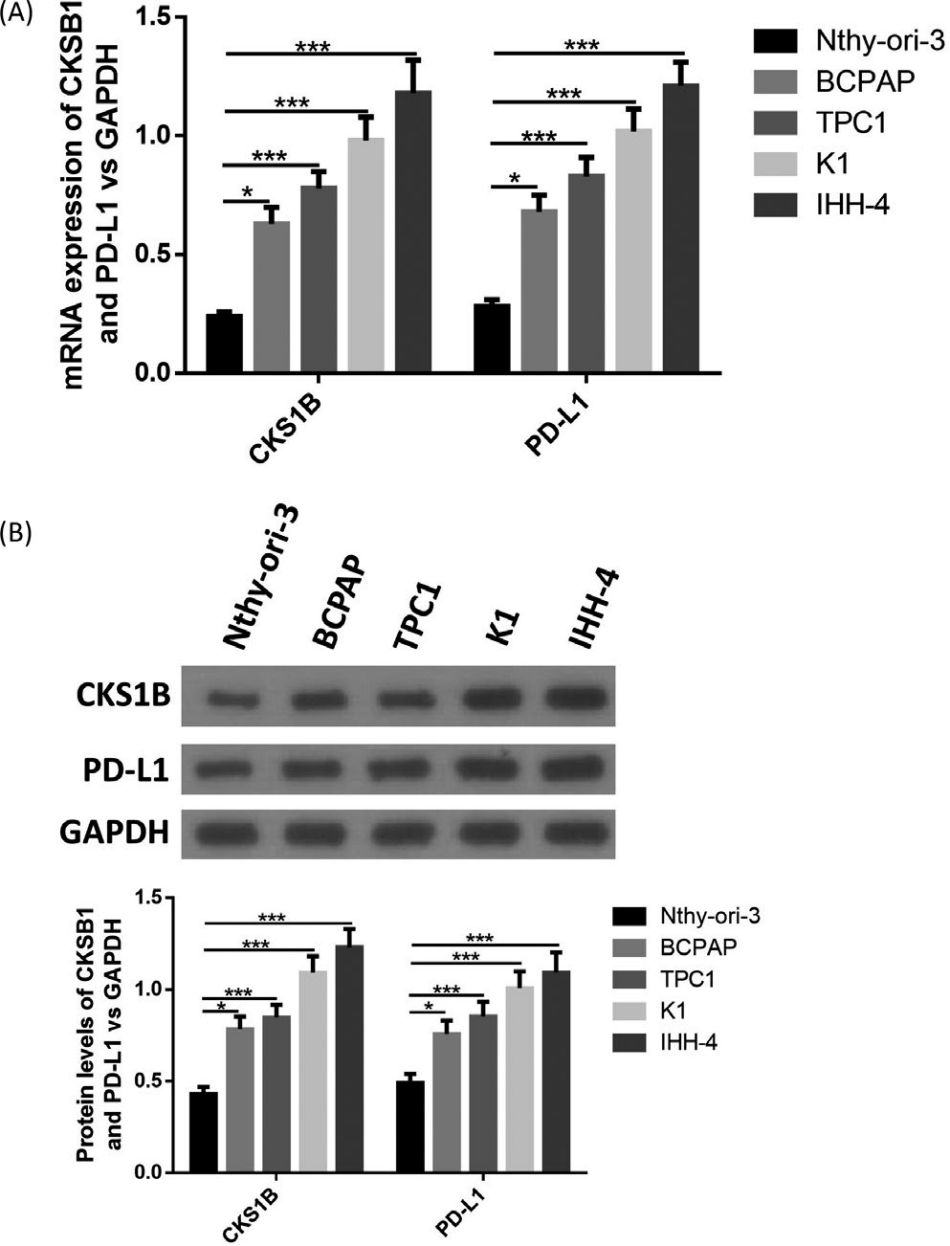
CKS1B and PD‐L1 were highly expressed in PTC cell lines. A, mRNA expression of CKS1B and PD‐L1 in PTC cell lines was determined by RT‐qPCR. B, Protein levels of CKS1B and PD‐L1 in PTC cell lines were determined by Western blotting. *P < .05, ***P < .001

First, we determined expression of CKS1B and PD‐L1 in PTC cell lines. As shown in Figure 1A‐B, both protein levels and mRNA levels of CKS1B and PD‐L1 were remarkably up‐regulated in PTC cell lines compared with the normal Nthy‐ori‐3 cells, suggesting that CKS1 and PD‐L1 were abnormally expressed in PTC. Then, IHH‐4 cells with the highest CKS1B expression and BCPAP cells with the lowest CKS1B expression in PTC cell lines were used for further researches.

### 3.2 Knockdown of CKS1B inhibited cell viability and invasion of PTC cells

**Figure 2:**
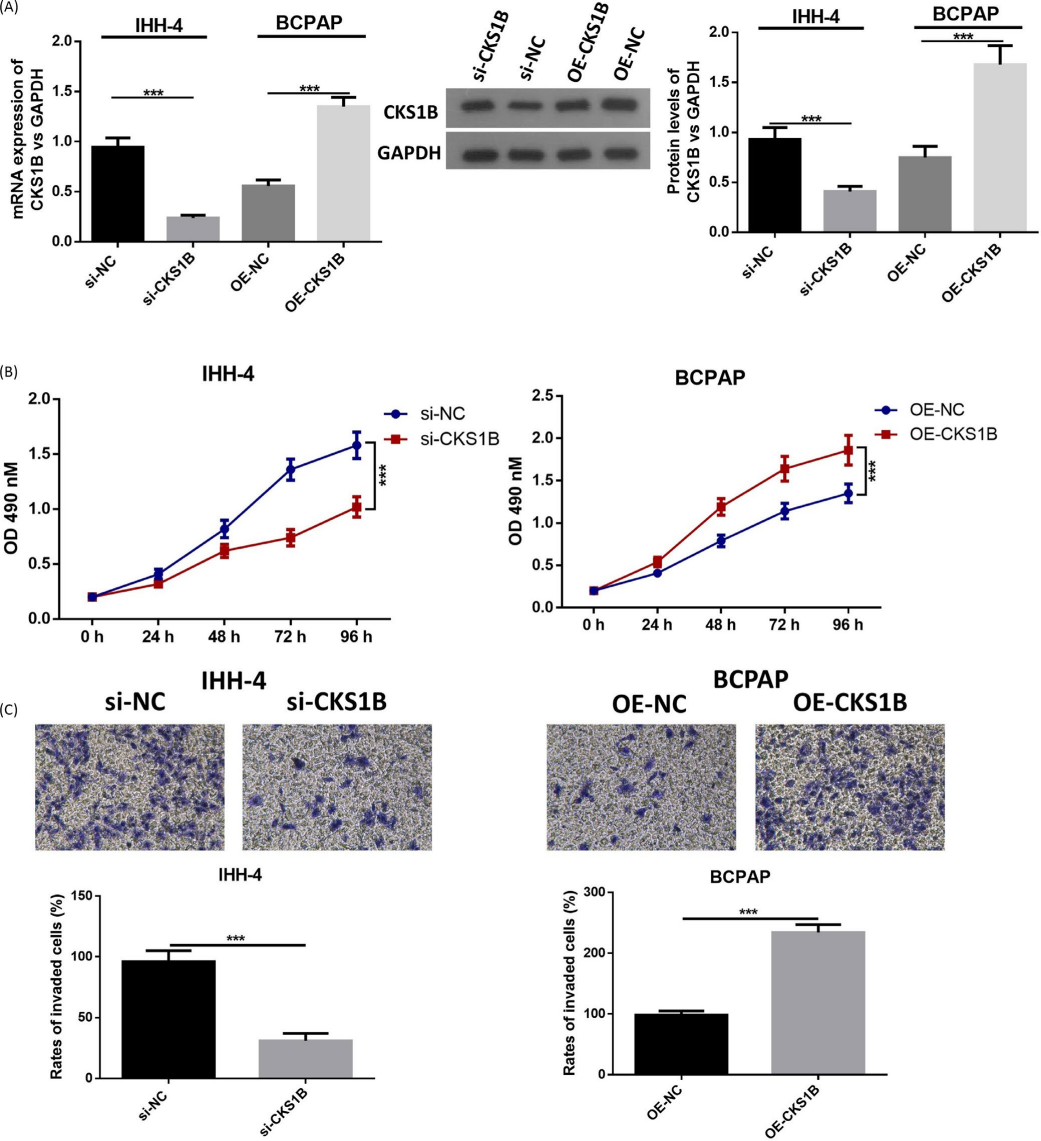
Knockdown of CKS1B inhibited cell viability and invasion of PTC cells. A, mRNA expression of CKS1B in cells transfected with si‐CKS1B or OE‐CKS1B. B, Cell viability of different groups of cells was measured by MTT assay. C, Cell invasion ability of different groups of cells was detected by transwell assay, Magnificationx100. ***P < .001

To further investigate role of CKS1B in PTC development, CKS1B was overexpressed in BCPAP cells by transfection with OE‐CKS1B plasmids and was knocked down in IHH‐4 cells by transfection with si‐CKS1B. After successful transfection (Figure 2A), MTT assay showed cell viability was dramatically suppressed by transfection with si‐CKS1B and was remarkably enhanced by transfection with OE‐CKS1B (Figure 2B). Meanwhile, knockdown of CKS1B by si‐CKS1B significantly reduced the invaded and migrated cell ratios than the NC cells, and overexpression of CKS1B by transfecting OE‐CKS1B led to opposite results (Figure 2C). All these results indicated that CKS1B promoted cell proliferation and invasion of PTC cells and inhibition of CKS1B suppressed these effects.

### 3.3 Knockdown of CKS1B inhibited STAT3/PD‐L1 signaling and Akt phosphorylation in PTC cells

**Figure 3:**
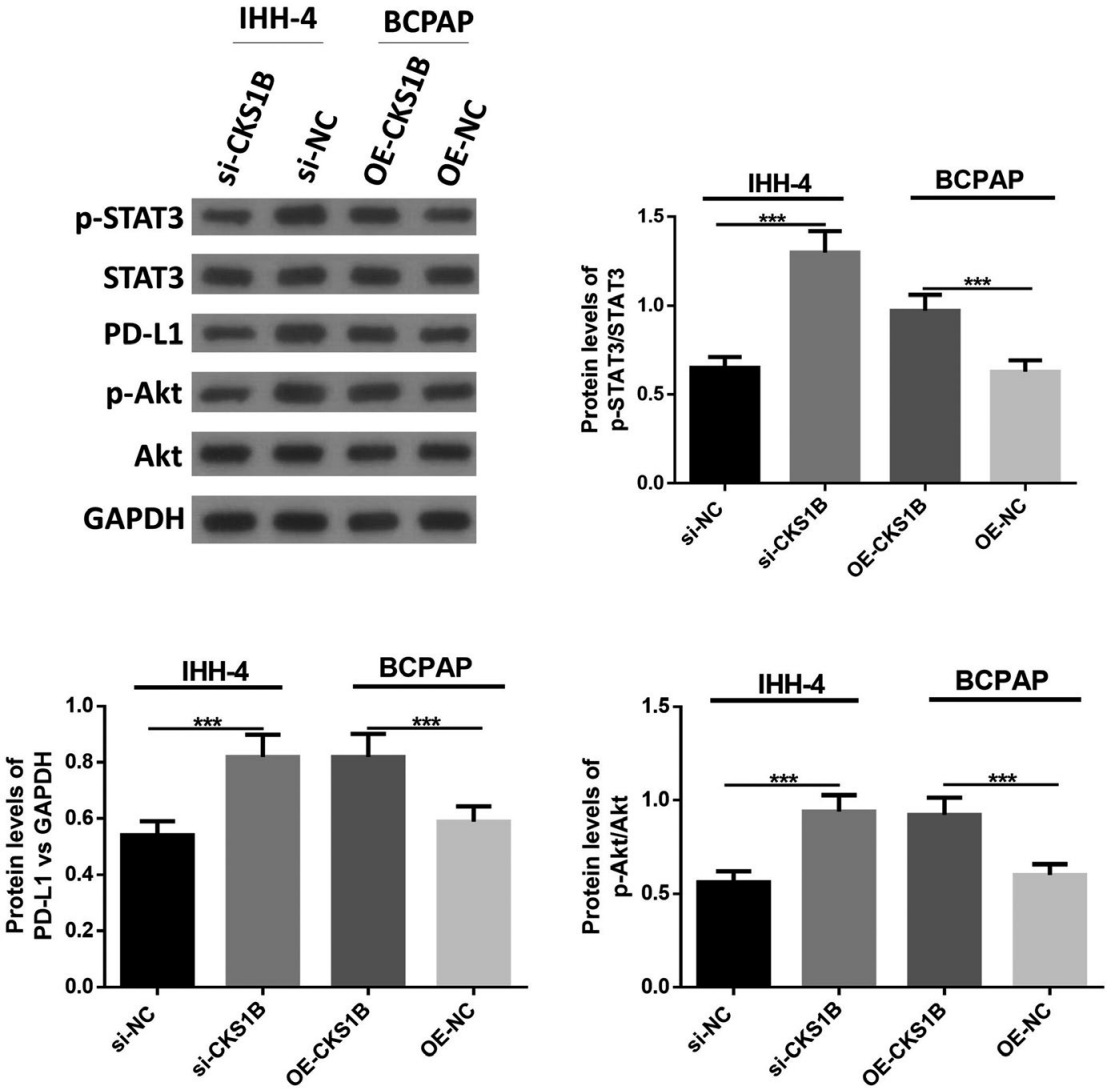
Knockdown of CKS1B inhibited STAT3/PD‐L1 signaling and Akt phosphorylation in PTC cells. Protein levels of p‐STAT3, STAT3, PD‐L1, p‐Akt, and Akt were measured by Western blotting. ***P < .001

Then, expression of p‐STAT3, STAT3, p‐Akt, Akt, and PD‐L1 was measured in IHH‐4 and BCPAP cells with overexpressed or suppressed CKS1B. It was observed that the knockdown of CKS1B remarkably decreased the protein levels of p‐STAT3, p‐Akt and PD‐L1, while overexpression of CKS1B resulted in increased protein levels of the above factors (Figure 3), suggesting knockdown of CKS1B might regulate cell function of PTC cell lines through regulation of STAT3/PD‐L1 signaling and Akt phosphorylation.

### 3.4 Inhibition of STAT3 or PD‐L1 reversed the effects of overexpressed CKS1B on PTC cells

**Figure 4:**
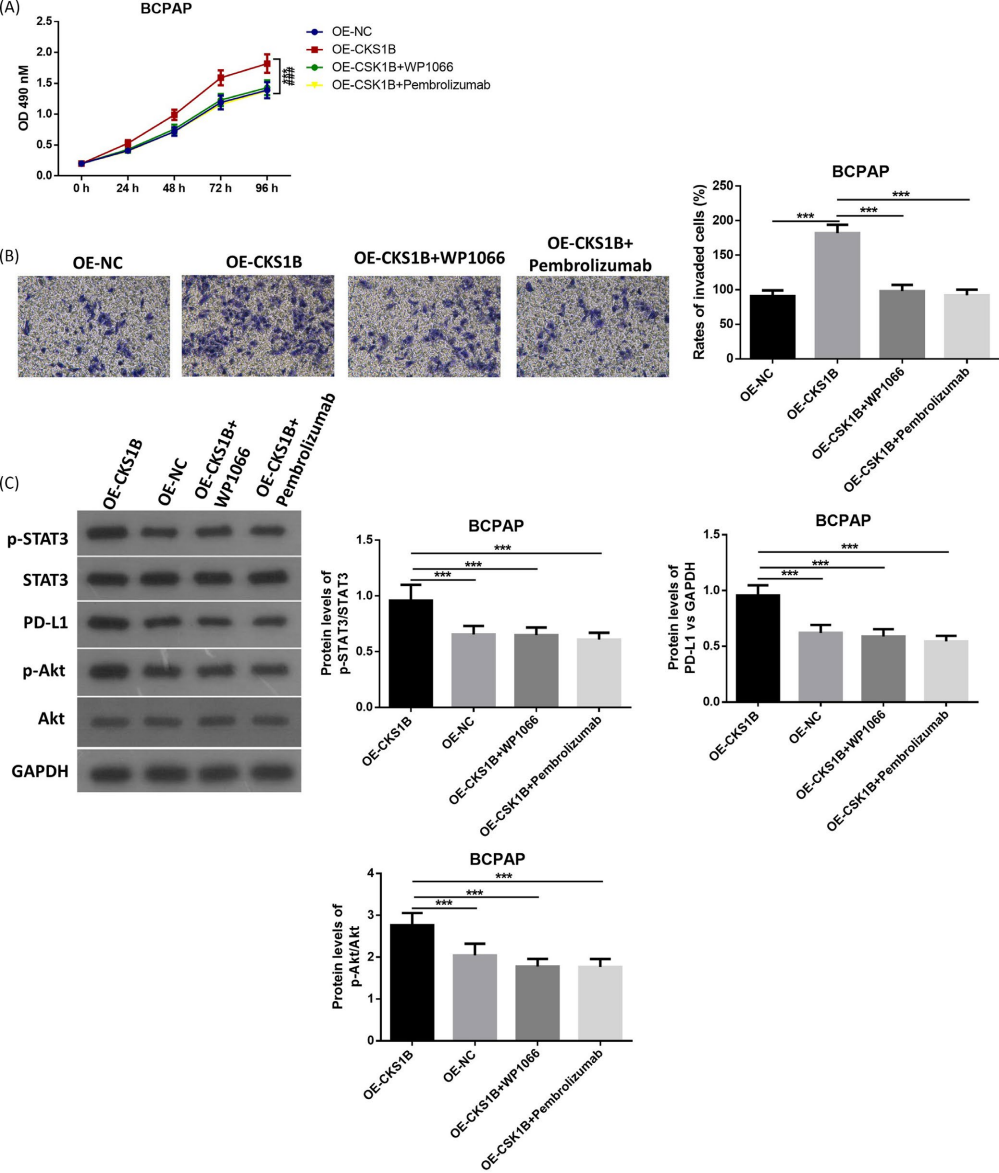
Inhibition of STAT3 reversed the effects of overexpressed CKS1B on PTC cells. A, Cell viability of different groups of cells was measured by MTT assay. B, Cell invasion ability of different groups of cells was detected by transwell assay, Magnificationx100. C, Protein levels of p‐STAT3, STAT3, PD‐L1, p‐Akt, and Akt were measured by Western blotting. ***P < .001

At last, we inhibited STAT3 signaling by treatment of WP1066 and inhibited PD‐L1 by treatment of Pembrolizumab. As shown in Figure 4A‐B, when CKS1B was overexpressed, cell viability was significantly promoted, as well as cell invasion of BCPAP cells. However, either the inhibition of STAT3 or PD‐L1 remarkably reversed these effects by overexpression of CSK1B. Besides, inhibition of STAT3 or PD‐L1 also suppressed the up‐regulation of p‐STAT3, PD‐L1, and p‐Akt by overexpression of CKS1B (Figure 4C). All these results further suggested that CKS1B might regulate cell proliferation and invasion of PTC cells by regulation of STAT3/PD‐L1/p‐Akt signaling.

## 4 DISCUSSION

Although PTC is a relative less malignant tumor compared with other thyroid carcinoma such as medullary thyroid carcinoma and anaplastic thyroid carcinoma, the prognosis for recurrence patients and patients with long distant metastasis is still poor.^16^, ^17^ CKS1B is reported as a cancer promoter; however, its role in PTC is rarely noticed. In this research, we demonstrated for the first time that CKS1B was elevated in PTC cell lines and could promote PTC cell proliferation and invasion through regulation of STAT3/PD‐L1 signaling and Akt phosphorylation.

Role of CKS1B in cancer development has been reported in several studies. It was reported that down‐regulation of miR‐1258 promoted carcinogenesis and progression of hepatocellular carcinoma by activation of CKS1B.^18^ Besides, overexpression of CKS1B contributed to poor prognosis of nasopharyngeal carcinoma.^7^ It was also found that CKS1B could induce chemoresistance in lung cancer which could be reversed by 3‐O‐(Z)‐coumaroyloleanolic acid.^19^ In the present research, we also found that CKS1B was up‐regulated in PTC cells, which was consistent with the above previous researches.

The regulation effects of CKS1B on STAT3 signaling were also reported in many researches. Generally, CKS1B/STAT3 axis accounts for cancer development. In an early study, Zhan et al showed CKS1B contributed to myeloma cell viability by activation of JAK/STAT3 signaling.^20^ CKS1B could also activate STAT3 signaling in multiple myeloma by promoting the degradation of p21.^21^ Another study showed elevated CKS1B promoted myeloma cell drug resistance by activation of STAT3 signaling.^13^ In the present study, we observed that CKS1B could also positively regulate STAT3/PD‐L1 signaling in PTC cells, and the inhibition of CKS1B could suppress cancer development of PTC by regulating STAT3/PD‐L1 signaling.

PD‐L1 has been noticed in PTC development in recent years. It was found that PD‐L1 was up‐regulated in PTC patients and patients with higher PTC level had higher risk for recurrence.^22^ Another study also showed GKS1B/STAT3 axis could enhance the expression of PD‐L1 and further promote cancer development of lung cancer cells.^12^ Besides, the positive interaction between PD‐L1 and Akt has also been reported in several cancers such as lung cancer ^23^ and lymphoma.^24^ In another research, Abdelhamed et al found Akt/STAT3 signaling could regulate PD‐L1 expression in non‐small cell lung cancer.^25^ In our research, we also found that activation of STAT3 by CSK1B could both facilitate the expression of PD‐L1 and the phosphorylation of Akt.

The present study also has some limitations. First, how CSK1B interacts with STAT3 and PD‐L1 is still unclear, and the interaction between STAT3 and PD‐L1 also needs to confirm. Secondly, whether other signaling pathways are involved in this process is also unknown. Thirdly, the role of Akt in GKS1B/STAT3/PD‐L1 axis is not clearly defined. All these need more studies to illuminate.

In conclusion, we investigated the role of CSK1B and its relationship between STAT3/PD‐L1 signaling and Akt phosphorylation in PTC. We found the inhibition of CSK1B could suppress cell viability and invasion of PTC cells through inhibition of STAT3/PD‐L1 signaling and Akt phosphorylation. This study might provide more insights into CSK1B/STAT3/PD‐L1 in PTC development.
